# Angiogenesis genotyping and clinical outcome during regorafenib treatment in metastatic colorectal cancer patients

**DOI:** 10.1038/srep25195

**Published:** 2016-04-27

**Authors:** Riccardo Giampieri, Lisa Salvatore, Michela Del Prete, Tiziana Prochilo, Marco D’Anzeo, Cristian Loretelli, Fotios Loupakis, Giuseppe Aprile, Elena Maccaroni, Kalliopi Andrikou, Maristella Bianconi, Alessandro Bittoni, Luca Faloppi, Laura Demurtas, Rodolfo Montironi, Marina Scarpelli, Alfredo Falcone, Alberto Zaniboni, Mario Scartozzi, Stefano Cascinu

**Affiliations:** 1 Medical Oncology, Translational Research Unit, Azienda Ospedaliero-Universitaria Ospedali Riuniti, Università Politecnica delle Marche, Ancona, Italy; 2 Medical Oncology, Azienda Ospedaliero-Universitaria Pisana, Università di Pisa, Pisa, Italy; 3 Medical Oncology, Fondazione Poliambulanza Brescia, Brescia, Italy; 4 Medical Oncology, Azienda Ospedaliera di Udine, Università di Udine, Udine, Italy; 5 Pathology, Azienda Ospedaliero-Universitaria Ospedali Riuniti, Università Politecnica delle Marche, Ancona, Italy; 6 Medical Oncology, Azienda Ospedaliera Universitaria di Cagliari, Università di Cagliari, Cagliari, Italy

## Abstract

Regorafenib monotherapy is a potential option for metastatic colorectal cancer patients. However, the lack of predictive factors and the severe toxicities related to treatment have made its use in clinical practice challenging. Polymorphisms of VEGF and its receptor (VEGFR) genes might regulate angiogenesis and thus potentially influence outcome during anti-angiogenesis treatment such as regorafenib. Aim of our study was to evaluate the role of VEGF and VEGFR genotyping in determining clinical outcome for colorectal cancer patients receiving regorafenib. We retrospectively collected clinical data and samples (tumour or blood) of 138 metastatic colorectal cancer patients treated with regorafenib. We analysed the correlation of different VEGF-A, VEGF-C and VEGFR-1,2,3 single nucleotide polymorphisms (SNPs) with patients’ progression-free survival (PFS) and overall survival (OS). Results from angiogenesis genotyping showed that only VEGF-A rs2010963 maintained an independent correlation with PFS and OS. Among clinical factors only ECOG PS was independently correlated with OS, whereas no correlation with PFS was evident. Grouping together those results allowed further patients stratification into 3 prognostic groups: favourable, intermediate and unfavourable. VEGF-A rs2010963 genotyping may represent an important tool for a more accurate selection of optimal candidates for regorafenib therapy.

Regorafenib is a potential option in heavily pre-treated metastatic colorectal cancer patients. This drug has proven its efficacy in the CORRECT and CONCUR trials^1^,^2^ as well as in the observational analyses CONFIRM and REBECCA^3^,^4^.

Available data suggest that only approximately 50% of patients treated might obtain a clinical benefit from regorafenib in the face of a not negligible proportion of potentially harmful side effects. Therefore the identification of patients who are more likely to benefit from this treatment would also spare unnecessary side effects in those who do not gain any clinically meaningful benefit from the drug.

Differently from anti-EGFR monoclonal antibodies for which we have a well-established selection criterion with the evaluation of RAS mutational status (K-ras, N-ras: rat sarcoma viral oncogene homologous; K stands for Kirsten, N stands for Neural)^5^,^6^, results for anti-angiogenic treatment options, including regorafenib, are still conflicting^7^. The inhibition of tumour angiogenesis, primarily based on the vascular endothelial growth factor (VEGF)-driven pathway, is one of the primary mechanisms of action of regorafenib treatment. A growing body of evidence suggested a possible correlation between an altered expression of the angiogenetic pathway identified through polymorphisms analysis and global outcome in colorectal, gastric, breast, ovarian, renal cell and hepatocellular patients treated with drugs directed against tumour neoangiogenesis^8^,^9^. Remarkably all known VEGF polymorphisms are not located in the coding region, therefore different biological mechanisms in influencing gene expression and then clinical outcome have been proposed^10^,^11^. Many molecular events ultimately leading to tumour neoangiogenesis have been in fact linked to SNPs in the VEGF and VEGF receptor (VEGFR) genes. Several SNPs in the promoter, 5- and 3-untranslated regions (UTRs) are present in the VEGF family.

In the present analysis we evaluated the potential role of VEGF and VEGFR polymorphisms in regorafenib treated colorectal cancer patients in order to define specific subgroups more likely to benefit from such a treatment approach.

## Results

### Patients’ characteristics

One hundred and thirty-eight patients were eligible for analysis: 76 males (55%) and 62 females (45%), median age was 63 years (33–78). Seventy patients (51%) were K-RAS or RAS wild type. Most of the patients had an ECOG PS (Eastern Cooperative Oncology Group-Performance Status) 0 at the time of start of regorafenib (84%) and the majority of them received at least 4 previous lines of treatment (96 patients, 70%).

In the whole population median OS was 7.3 months while median PFS was 2.4 months. Forty-two patients (30%) obtained disease stabilization whereas the remaining 96 patients (70%) progressed under treatment. Partial or complete responses were not observed in this group of patients.

### Results of angiogenesis genotyping and clinical factors analysis

All SNPs genotyped presented an overall call rate of ≥90%. We have evaluated concentration and purity index of each sample by UV spectrophotometry as the ratio absorbance 260/280 nm. All samples presented a purity index between 1.5 and 2.0. The frequencies of the tested genotypes resulted comparable to those reported in Caucasians, with no significant deviation from the Hardy–Weinberg equilibrium. Linkage disequilibrium (LD) was not observed.

Only VEGF-A rs2010963 (+405G > C) was associated with both OS and PFS. Twenty patients (14%) presented the CC homozygous genotype, whereas the remaining 118 patients (86%) harbored the GG or GC genotype.

**Figure 1:**
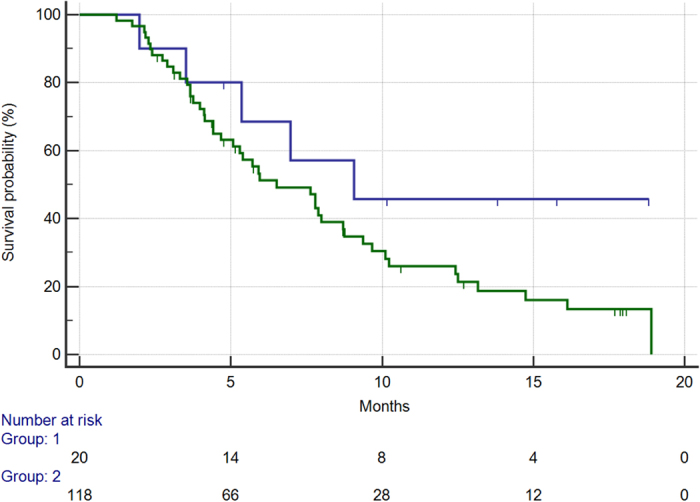
Overall survival for patients with CC rs2010963 SNP (405 G->C) genotype (BLUE) vs patients with either GC or GG genotype (GREEN) (9.0 vs 6.5 months, HR: 0.52, 95%CI: 0.34–0.99, p = 0.04).

In this group of patients median OS was 9 months vs. 6.5 months for CC homozygous patients vs. patients showing the GG or GC genotype (hazard ratio HR: 0.52, confidence interval 95%CI: 0.34–0.99, p = 0.049) [Fig. 1].

**Figure 2:**
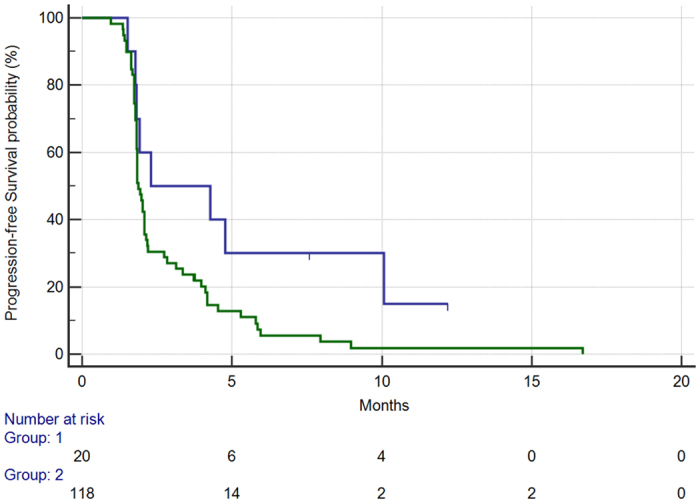
Progression-free survival for patients with CC rs2010963 SNP (405 G->C) genotype (BLUE) vs. patients with either GC or GG genotype (GREEN) (2.2 vs. 1.8 months, HR: 0.49, 95%CI: 0.33–0.81, p = 0.003).

Median PFS in patients with the CC genotype vs. the remaining patients (GG or GC genotypes) was respectively 2.2 vs. 1.8 months (HR: 0.49, 95%CI: 0.33–0.81, p = 0.0038) [Fig. 2].

In the 20 patients harboring the CC genotype, 11 (55%) patients obtained disease stabilization while 9 patients (45%) progressed under treatment. In the remaining 118 patients 31 (26%) experienced disease stabilization and the remaining 87 progressed while on treatment (74%). This difference in the disease control rate (DCR) was statistically significant (p = 0.02).

VEGF-R2 rs1870377 and VEGF-R3 rs307805 were both correlated to OS (but not PFS), whereas VEGF-R1 rs664393 resulted linked to PFS only. Table 1 summarizes survival outcomes according to all polymorphisms tested.

**Figure 3:**
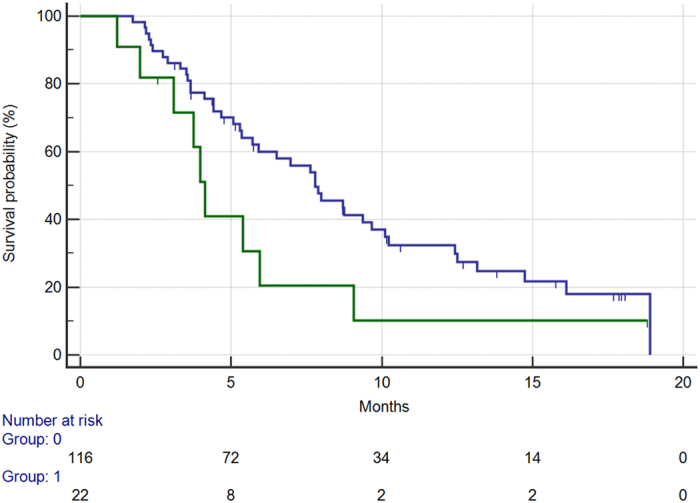
Overall survival for patients with ECOG:0 status (BLUE) vs patients with ECOG:1 or more status (GREEN) (7.8 months vs 4.1 months, HR: 0.52, 95%CI:0.21–0.81, p = 0.009).

**Figure 4:**
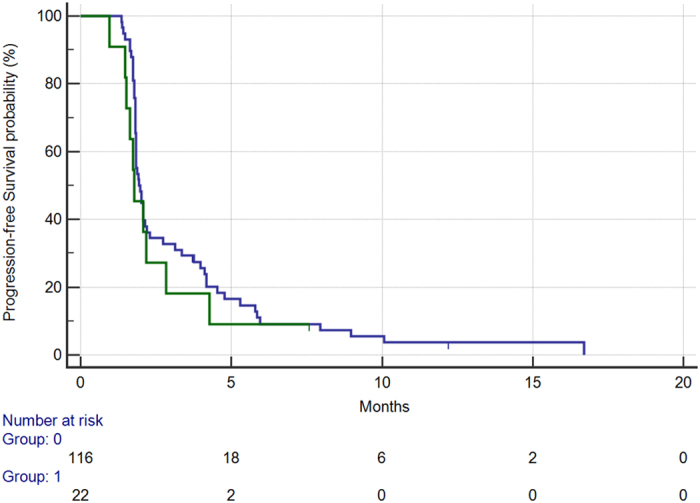
Progression-free survival for patients with ECOG:0 status (BLUE) vs patients with ECOG:1 or more status (GREEN) (1.9 vs 1.7 months, HR: 0.75, 95%CI: 0.41–1.24, p = 0.23).

Among clinical factors tested, ECOG PS resulted significantly associated with worse overall survival. In particular, in the 22 patients (16%) presenting with ECOG PS ≥ 1 vs. those presenting with an ECOG PS 0 (116, 84%) median overall survival was respectively 4.1 months vs. 7.8 months (HR: 0.52, 95%CI: 0.21–0.81, p = 0.009) [Fig. 3]. Apparently ECOG PS was not able to influence PFS (median PFS 1.7 months vs. 1.9, HR: 0.75, 95%CI: 0.41–1.24, p = 0.23) [Fig. 4].

At multivariate analysis VEGF-A rs2010963 (p = 0.009) and ECOG performance status (p = 0.004) demonstrated an independent role as predictive factors for overall survival whereas VEGF-R3 rs307805 and VEGF-R2 rs1870377 resulted not significant. In particular, Exp(B) for ECOG performance status was 2.80 (95%CI:1.59–4.92) whereas Exp(B) for rs2010963 was 2.77 (95%CI:1.37–5.60).

On the contrary only VEGFA rs2010963 resulted independently correlated to PFS (p = 0.005). Major clinical characteristics according to VEGFA rs2010963 resulted comparable across different genotypes and to the whole patients population [Table 2].

**Figure 5:**
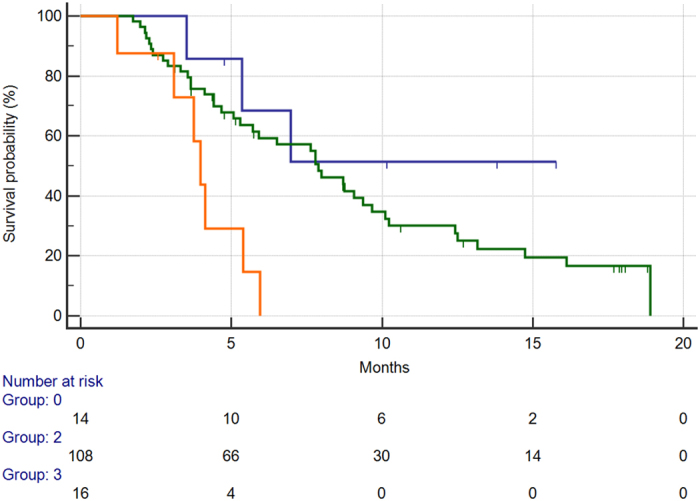
Median overall survival for patients in the favourable group (VEGF-A rs2010963 CC genotype SNP and ECOG PS 0) (BLUE) vs. patients in the intermediate group (either VEGF-A rs2010963 CC or ECOG PS 0 status (GREEN) vs. patients in the unfavourable group (VEGF-A rs2010963 CG or GG genotype and ECOG PS ≥1) (ORANGE) (NR vs. 7.8 months vs. 3.9 months, p < 0.0001).

Taken together ECOG performance status and VEGFA rs2010963 allowed a further patient stratification into 3 groups with different clinical outcome during regorafenib: favourable group (both favourable VEGFA rs2010963 genotype and ECOG PS 0), intermediate group (either favourable VEGFA rs2010963 genotype or ECOG PS 0) and unfavourable group (both unfavourable VEGFA rs2010963 genotype and ECOG PS ≥1). Fourteen patients (10%) were allocated in the favourable group, 108 patients (78%) were allocated in the intermediate group and 16 patients (12%) were allocated in the unfavourable group. Median OS resulted progressively decreased across these groups (OS not reached, 7.8 and 3.9 months respectively in the favourable, intermediate and unfavourable group, p < 0.0001) [Fig. 5].

**Figure 6:**
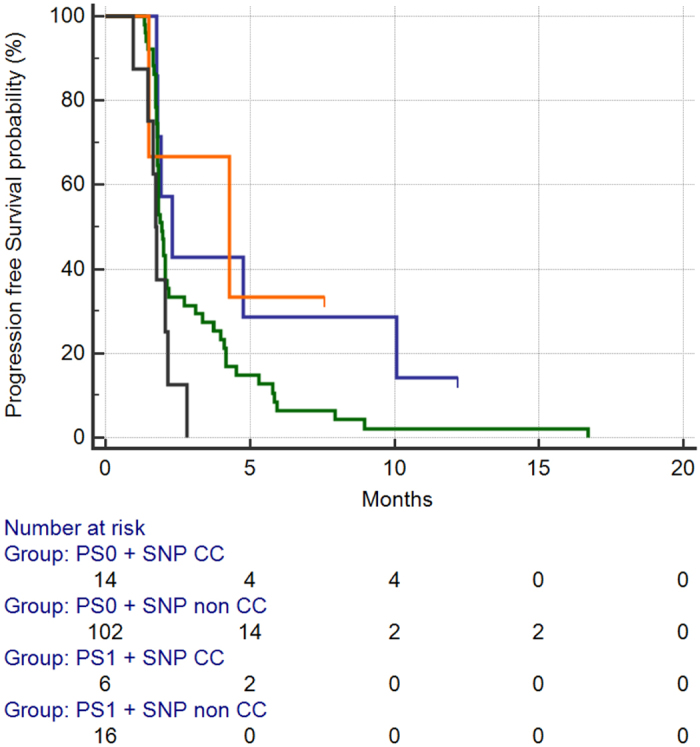
Median PFS for patients stratified accordingly to 4 different subgroups: PS0 and CC homozygous subtype (BLUE) vs. PS0 and GC or GG homozygous subtype (GREEN) vs. PS1+ and CC homozygous subtype (ORANGE) vs. PS1+ and GC or GG homozygous subtype (BLACK).

**Figure 7:**
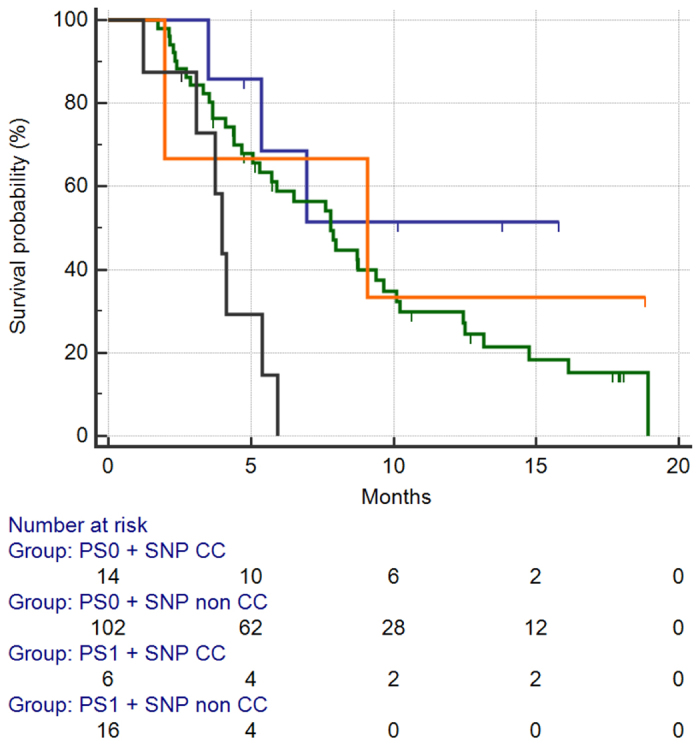
Median OS for patients stratified accordingly to 4 different subgroups: PS0 and CC homozygous subtype (BLUE) vs. PS0 and GC or GG homozygous subtype (GREEN) vs. PS1+ and CC homozygous subtype (ORANGE) vs. PS1+ and GC or GG homozygous subtype (BLACK).

We also analysed the relative impact of each of the two factors on survival: in particular, patients were divided into 4 groups (ECOG PS 0 and CC genotype vs. ECOG PS 0 and GC or GG genotype vs. ECOG PS 1 or higher and the CC genotype vs. ECOG PS 1 or higher and the GC or GG genotype). When we analysed the impact of the four different groups on PFS, patients with the CC genotype, regardless of performance status, showed similar (and statistically comparable, HR:0.86, 95%CI:0.3834–1.9530) [Fig. 6]. On the contrary overall survival analysis showed that patients with ECOG PS 1 higher and the GC or GG genotype (16/138, 11%) had the worst survival among the 4 subgroups, whereas patients with ECOG PS 1 or higher and the CC genotype had comparable survival to patients with ECOG PS 0 and the GC or GG genotype [Fig. 7].

## Discussion

The introduction of regorafenib for the treatment of heavily pre-treated metastatic colorectal cancer undoubtedly filled the clinical gap caused by the previous lack of valid options in this setting. Furthermore, the concept of sustained angiogenesis inhibition in different lines of treatment for metastatic colorectal cancer patients who are not amenable to radical surgical resection proves to be effective in a series of already published works^12^,^13^,^14^,^15^.

The virtual absence of predictive factors for efficacy/resistance further restrained wide use of this treatment opportunity. The capability of regorafenib to pharmacologically interact with tumour-driven angiogenesis seems to indicate that this biological pathway may be a potentially relevant predictive factor for clinical outcome during therapy. However, recent analyses conducted on the cohort of patients enrolled in the CORRECT trial^16^ seemed to rule out a potential predictive role of soluble serum concentrations of various factors linked to neoangiogenesis (such as sVEGFR-1, VEGF-A, VEGF-C, angiopoietin-2 and others).

In our analysis we suggest that single nucleotide polymorphisms in the VEGF associated pathway may be linked to clinical outcome in patients treated with regorafenib. In particular the VEGFA rs2010963 resulted independently associated with median OS and PFS and disease control rate thus suggesting a possibly effective way for patients selection in a non-negligible proportion of cases. The VEGF-A rs2010963 CC genotype that resulted linked to improved outcome is present in 14% of the population analysed.

The VEGF-A rs2010963 is located in the 5′-UTR region, nearby the gene promoter region thus potentially influencing VEGF-A expression and resulting in an increased tumour angiogenesis. Many studies confirmed in fact a central role for the VEGF-A rs2010963 in biological systems either as a risk factor for human carcinogenesis or predictive factor for anti-angiogenesis therapy^17^.

On the other hand different studies investigated the impact of VEGF-A rs2010963 on clinical outcome during anti-angiogenesis therapy. According to present results, in advanced HCC receiving sorafenib patients harbouring the VEGF-A rs2010963 CC genotype showed a significantly improved overall and progression free survival^9^.

Whether VEGF-A rs2010963 in our analysis may represent a prognostic or predictive factor is matter of debate. On the one hand the absence of a control group make this distinction very challenging, but on the other hand the impact on PFS and DCR seems to indicate a predictive more than a prognostic role.

The ECOG PS is an important prognostic indicator and a powerful driver for strategy decision in advanced colorectal cancer patients. In our analysis patients presenting with an ECOG PS 0 seemed to benefit the most from regorafenib treatment. However in this case the ECOG PS did not correlate with PFS or DCR thus suggesting a more pronounced prognostic impact. Interestingly patients with ECOG PS 1 or higher showing the rs2010963 CC polymorphism still demonstrated an improved prognosis compared with those with ECOG PS 1 or higher, but who had the rs2010963 GC or GG polymorphism. We can then speculate that in patients harbouring the rs2010963 CC polymorphism the improvement in prognosis seemed independent from ECOG PS and hypothetically related to a true susceptibility of the tumour to regorafenib therapy.

Accordingly at the multivariate analysis for overall survival both factors (ECOG PS and rs2010963 polymorphisms) showed not only an independent role, but also a comparable influence in determining overall survival (as it can be assumed by the similar results of Exp(B) at multivariate analysis for both factors).

Coupling results from VEGF-A rs2010963 and ECOG PS led us to further characterize different patients’ groups with different outcomes during regorafenib.

In particular patients showing an unfavourable profile (both unfavourable VEGFA rs2010963 genotype and ECOG PS ≥1) seemed to derive little or no benefit from the use of regorafenib. In this group of patients a more than usual careful evaluation of both the risk-to-benefit ratio and patients expectations from therapy should drive the treatment decision.

There is a series of considerations that have to be made regarding the limits of our analysis: the retrospective nature of our analysis, in absence of a “control group” who did not receive regorafenib, is unable to fully establish the prognostic or truly predictive (or perhaps both) nature of the markers used.

Another important consideration is that, when looking at the result concerning progression-free survival, even though median progression free survival was significantly different between patients with favourable VEGF-A rs2010963 genotype when compared with the other subset (2.2 vs. 1.8 months), the difference in the risk of progression was truly evident only after 2 months of observation time, with about 40% of patients who directly progressed at the first radiological evaluation regardless of their VEGF genotype profile: this fact suggests that factors other than VEGF pathway genotyping may be crucial in determining the efficacy of regorafenib treatment.

A further relevant consideration is about the relatively small number of patients who would be allocated into either prognostic group (favourable and unfavourable) according to our findings. In particular, patients with the favourable subgroup would represent about 10% of the patients treated, compared with 10–12% of patients in the unfavourable subgroup.

In clinical trials investigating regorafenib monotherapy for metastatic colorectal cancer patients, the proportion of patients who truly benefited from treatment (i.e. with a PFS longer than 6 months) was about 10–12%^1^,^2^,^3^. At the same time, the proportion of patients showing a PFS shorter than 2 months in all these trials taken together is once again about 10–12%. These proportions are strikingly similar to those observed in our analysis, thus suggesting that we could individuate 3 groups of patients among those treated with regorafenib. A small number of patients deriving the highest benefit from the drug with the longest progression free survival, a further small group of patients who does not derive any clinical benefit from treatment and, due to rapid disease progression, usually stops treatment very soon and progresses shortly thereafter, and, last, the rest of the patients with intermediate characteristics.

We believe that after validation in a larger, hopefully prospective, series of metastatic colorectal cancer patients, VEGF-A rs2010963 may represent an important tool for a more accurate selection of patients potentially candidates for regorafenib. This selection opportunity will ultimately improve the therapeutic index of such a treatment approach by limiting treatment to potentially responding patients and sparing unnecessary toxicity to those unlikely to benefit.

## Methods

### Patients Selection

The ALICE-3 (Angiogenesis lInked coLorectal CancEr) is a retrospective multicentre study. All consecutive histologically-proven metastatic colorectal cancer patients, previously treated with all standard chemotherapy-regimens (oxaliplatin, irinotecan, 5FU, bevacizumab and either cetuximab or panitumumab in case of K-RAS, and then RAS, wild type status) receiving regorafenib monotherapy were eligible for our analysis.

All patients started treatment with regorafenib at the dose of 160 mg/die (day 1 to 21 every 28 days), dose reduction was applied as clinically indicated. Tumour response was evaluated every 8 weeks by clinicians’ assessment and according to the response evaluation criteria for solid tumours (RECIST). Written informed consent was obtained for all patients enrolled into the analysis and the study was approved by the Ethical Committee of the Azienda Ospedaliera Universitaria of Ancona, Italy. The methods were carried out in accordance with the approved guidelines.

### VEGF and VEGFR genotyping

VEGF and VEGFR genotyping was performed using DNA extracted from formalin-fixed paraffin-embedded tissue blocks of metastatic colorectal carcinoma or from whole blood.

For tissue blocks, paraffin wax was removed with xylene and the samples were washed twice with 100% ethanol. DNA was isolated from the deparaffinised tissue using the RecoverAll™ Total Nucleic Acid Isolation Kit for (formalin fixed paraffin embedded) Tissues (Applied Biosystems, Foster City, CA, USA), according to the manufacturer’s instructions. DNA from each sample was then eluted in 120 μl of eluting solution.

For blood samples genomic DNA was extracted from 2 ml of whole blood by FlexiGene DNA kit (Qiagen Inc., Valencia, CA, United States), following the manufacturer’s instructions. Concentration and purity index of each sample were evaluated by UV spectrophotometry as the ratio absorbance 260/280 nm; a purity index of 1.5–2.0 was considered optimal.

Single nucleotide polymorphisms (SNPs) within each gene were selected using the Pupasuite software (http://pupasuite.bioinfo.cipf.es/index.jsf - version 2.0.0, bioinfo 2008), the CIPF (Centro de Investigacion Principe Felipe) Single Nucleotide Polymorphism database (dbSNP) generated by the National Centre for Biotechnology Information (http://www.ncbi.nlm.nih.gov/SNP) and by review of the medical literature, using the following criteria:

1. The polymorphism had some degree of likelihood to alter the structure or the expression of the gene in a biologically relevant manner;
2. The minor allele frequency was above 10% (with the only exception of rs2305948, rs6877011 and rs307822);
3. The genetic polymorphism was established and well-documented.

Further considerations led the selection of SNPs for our study. A correlation between the presence of a specific allele on a polymorphic site and the expression of the respective protein has been previously documented for VEGF.

Selected SNPs, chromosomal locations, positions and biological effects are summarised in Table 3.

SNP genotyping was performed by TaqMan technology, using SNP genotyping assay (Applied Biosystems, Foster City, CA). Polymerase chain reaction (PCR) was performed and genotypes were analysed on the 7300 Real-Time PCR System (Applied Biosystems, Foster City, CA) using an ABI Prism 7300 Sequence Detection System software (version 1.3.1, Applied Biosystems, Foster City, CA). Each reaction contained 0.2 μl of total genomic DNA. Laboratory personnel blinded to patient status performed Genotyping, and a random 10% of the samples were repeated to validate genotyping procedures. All SNPs genotyped had to present an overall call rate of ≥90% to be included in our analysis, all samples resulted significant during the analysis and didn’t need test repetition.

### Data Management and statistical Analysis

To detect a difference in the effect size (HR) with statistical significance in the proportion of patients surviving at six months according to genotyping and assuming a 6-month overall survival (OS) of 50% in an unselected regorafenib treated population and a 70% 6-month OS as a target, with α = 0.05 and β = 0.10, at least 121 patients were needed.

Statistical analysis was performed with MedCalc Statistical Software version 14.10.2 (MedCalc Software bvba, Ostend, Belgium; http://www.medcalc.org; 2014).

The association between categorical variables was estimated by Fisher exact test for categorical binomial variables or by chi-square test for all other instances. Survival probability over time was estimated by the Kaplan–Meier method.

Significant differences in the probability of survival between the strata were evaluated by log-rank test. Cox’s multiple regression analysis was used to assess the role of polymorphisms as prognostic factor adjusted for those variables resulted significant at univariate analysis. The Holm-Sidak correction^18^ was used to adjust the values for multiple comparisons. Tested variables included gender (male vs. female), median age (<70 vs. ≥70 years), K-RAS or RAS status (wild type vs. mutant), ECOG performance status (0 vs. ≥1), previous lines for metastatic disease (≤3 vs. ≥4).

For statistical analysis, overall survival (OS) was defined as the time interval between the date of beginning of regorafenib treatment to death or last follow-up visit for patients who were lost at follow-up, whereas progression-free survival (PFS) was defined as the interval between the date of beginning of regorafenib treatment to death, first sign of clinical progression or last follow-up visit for patients who were lost at follow-up.

All genetic polymorphisms were examined for deviation from Hardy–Weinberg equilibrium using the Powermarker v.3.25 package (www.statgen.ncsu.edu/powermarker). LD analysis was also performed using the Powermarker v. 3.25 package. LD was estimated using r2, with r^2^ = 1 indicating complete LD and r^2^ = 0 indicating absent LD.

## Additional Information

**How to cite this article**: Giampieri, R. *et al.* Angiogenesis genotyping and clinical outcome during regorafenib treatment in metastatic colorectal cancer patients. *Sci. Rep.* **6**, 25195; doi: 10.1038/srep25195 (2016).

## Figures and Tables

**Table 1:** Impact of different genotypes on overall survival and progression free survival (in bold where statistically significant).

	PFS	p	OS	p
rs10434				
AA genotype (32, 23%)	1.82 months		6.51 months	
AG genotype (74, 54%)	2.01 months		7.89 months	
GG genotype (32, 23%)	1.92 months	0.14	4.67 months	0.21
rs2010963				
GG genotype (62, 44%)	**1.84 months**		**7.89 months**	
CG genotype (56, 41%)	**1.94 months**		**5.95 months**	
CC genotype (20, 15%)	**4.28 months**	**0.0098**	**9.08 months**	**0.049**
rs25648				
AA genotype (100, 72%)	1.94 months		6.51 months	
AT genotype (30, 22%)	1.91 months		7.99 months	
TT genotype (8, 6%)	2.02 months	0.96	9.38 months	0.18
rs3025039				
AA genotype (120, 87%)	1.91 months		7.63 months	
AT genotype (16, 12%)	3.12 months		5.72 months	
TT genotype (2, 1%)	1.84 months	0.24	3.55 months	0.06
rs699947				
CC genotype (44, 32%)	2.07 months		5.95 months	
AC genotype (56, 40%)	2.01 months		7.99 months	
AA genotype (38, 28%)	1.84 months	0.11	6.51 months	0.46
rs833061				
TT genotype (44, 32%)	2.07 months		5.95 months	
TC genotype (56, 40%)	2.01 months		7.99 months	
CC genotype (38, 28%)	1.84 months	0.11	6.51 months	0.46
rs4604006				
CC genotype (68, 49%)	1.84 months		7.8 months	
CT genotype (60, 43%)	1.97 months		5.95 months	
TT genotype (10, 8%)	3.75 months	0.50	13.16 months	0.34
rs7664413				
CC genotype (80, 58%)	1.84 months		7.8 months	
CT genotype (52, 38%)	1.97 months		5.95 months	
TT genotype (6, 4%)	3.75 months	0.41	NR	0.07
rs664393				
CC genotype (106, 77%)	**2.01 months**		6.97 months	
CT genotype (30, 22%)	**1.84 months**		8.72 months	
TT genotype (2, 1%)	**1.48 months**	**<0.0001**	5.95 months	0.77
rs7993418				
genotype (68, 49%)	1.84 months		7.8 months	
AG genotype (64, 46%)	1.97 months		6.51 months	
GG genotype (6, 5%)	5.3 months	0.42	10.23 months	0.32
rs1870377				
TT genotype (72, 52%)	1.97 months		**7.63 months**	
AT genotype (62, 45%)	1.84 months		**7.8 months**	
AA genotype (4, 3%)	2.12 months	0.98	**3.54 months**	**0.0061**
rs2071559				
AA genotype (44, 32%)	1.84 months		6.51 months	
AG genotype (56, 40%)	1.94 months		7.8 months	
GG genotype (38, 28%)	2.07 months	0.86	5.92 months	0.80
rs2305948				
CC genotype (114, 83%)	1.94 months		7.63 months	
CT genotype (24, 27%)	1.84 months		7.8 months	
TT genotype (0, 0%)	NA	0.88	NA	0.81
rs7667298				
CC genotype (40, 29%)	2.07 months		5.92 months	
CT genotype (62, 45%)	1.88 months		7.8 months	
TT genotype (36, 26%)	1.84 months	0.46	7.99 months	0.80
rs307805				
AA genotype (106, 77%)	1.91 months		**7.63 months**	
AG genotype (30, 22%	2.07 months		**8.75 months**	
GG genotype (2, 1%)	2.3 months	0.83	**3.52 months**	**0.0492**
rs6877011				
CC genotype (110, 80%)	1.97 months		6.97 months	
CG genotype (28, 20%)	1.86 months		7.8 months	
GG genotype (0, 0%)	NA	0.42	NA	0.48
rs307822				
CC genotype (104, 75%)	1.97 months		6.97 months	
CT genotype (34, 25%)	1.84 months		7.8 months	
TT genotype (0, 0%)	NA	0.08	NA	0.67

**Table 2:** Patients’ clinical characteristics according to rs2010963 genotyping.

	Total	rs2010963 CC homozygous	Rs2010963 GG homozygous - heterozygous	*p*
	138	20 (14%)	118 (86%)	
Sex				
Male	76 (55%)	10 (50%)	66 (56%)	
Female	62 (45%)	10 (50%)	52 (44%)	0.90
Median Age (range)	63 (33–78)	56 (42–71)	62 (33–78)	
≤70	110 (80%)	18 (90%)	92 (78%)	
>70	28 (20%)	2 (10%)	26 (22%)	0.34
K-RAS status				
Wild Type	70 (51%)	13 (65%)	57 (48%)	
Mutant	68 (49%)	7 (35%)	61 (52%)	0.25
ECOG PS				
0	116 (84%)	14 (70%)	102 (86%)	
≥1	22 (16%)	6 (30%)	16 (14%)	0.12
Previous treatment lines				
3 or less	42 (30%)	4 (20%)	38 (32%)	
4 or more	96 (70%)	16 (80%)	80 (68%)	0.40

**Table 3:** Chromosomal locations, positions and biological effects of investigated gene SNPs.

SNP ID	Gene	TaqMan catalog number	Chr	Chr. Position	Position in the gene/Effect	Codon exchange	aa. exchange
rs10434	VEGF-A	C_1647360_20	6	43753212	3′UTR(c)	–	–
rs2010963	VEGF-A	C_8311614_10	6	43738350	5′UTR(d)	–	–
rs25648	VEGF-A	C_791476_10	6	43738977	Syn(a); ESE(b)	TCC ⇒ TCT	S [Ser] ⇒ S [Ser]
rs3025039	VEGF-A	C_16198794_10	6	43752536	3′UTR(c)	–	–
rs699947	VEGF-A	C_8311602_10	6	43736389	Prom(e)	–	–
rs833061	VEGF-A	C_1647381_10	6	43737486	Prom(e)	–	–
rs4604006	VEGF-C	C_216109_10	4	177608775	Intronic	–	–
rs7664413	VEGF-C	C_11780939_10	4	177608707	Intronic	–	–
rs664393	VEGFR-1 FLT1	C_938327_20	13	29071001	3′UTR(c)	–	–
rs7993418	VEGFR-1 FLT1	C_1910654_10	13	28883061	Syn(a); ESE(b)	TAC ⇒ TAT	Y [Tyr] ⇒ Y [Tyr]
rs1870377	VEGFR-2 KDR	C_11895315_20	4	55972974	Missense	CAA ⇒ CAT	Q [Gln] ⇒ H [His]
rs2071559	VEGFR-2 KDR	C_15869271_10	4	55992366	Init. Transcription	–	–
rs2305948	VEGFR-2 KDR	C_22271999_20	4	55979558	Missense	GTA ⇒ ATA	V [Val] ⇒ I [Ile]
rs7667298	VEGFR-2 KDR	C_28992770_10	4	55991731	5′UTR(d)	–	–
rs307805	VEGFR-3 FLT4	C_918880_10	5	180077487	Prom(e); TFBS(g)	–	–
rs6877011	VEGFR-3 FLT4	C_29057584_10	5	180029471	3′UTR(c)	–	–
rs307822	VEGFR-3 FLT4	C_918831_1	5	180028717	3′UTR(c)	–	–

^(a)^Syn: Synonymous substitution.

^(b)^ESE: Exon Splicing Enhancer.

^(c)^3′UTR: Untranslated Region 3′UTR.

^(d)^5′UTR: Untranslated Region 5′UTR.

^(e)^Prom: Promoter region.

^(g)^TFBS: Predicted Trascription Factor Binding Site.
